# Drugs from nature targeting inflammation (DNTI): a successful Austrian interdisciplinary network project

**DOI:** 10.1007/s00706-015-1653-y

**Published:** 2016-02-25

**Authors:** Birgit Waltenberger, Atanas G. Atanasov, Elke H. Heiss, David Bernhard, Judith M. Rollinger, Johannes M. Breuss, Daniela Schuster, Rudolf Bauer, Brigitte Kopp, Chlodwig Franz, Valery Bochkov, Marko D. Mihovilovic, Verena M. Dirsch, Hermann Stuppner

**Affiliations:** Institute of Pharmacy/Pharmacognosy and Center for Molecular Biosciences Innsbruck (CMBI), University of Innsbruck, Innsbruck, Austria; Department of Pharmacognosy, University of Vienna, Vienna, Austria; Cardiac Surgery Research Laboratory, Department of Cardiac Surgery, Innsbruck Medical University, Innsbruck, Austria; Department of Vascular Biology and Thrombosis Research, Center of Physiology and Pharmacology, Medical University Vienna, Vienna, Austria; Institute of Pharmacy/Pharmaceutical Chemistry and CMBI, University of Innsbruck, Innsbruck, Austria; Institute of Pharmaceutical Sciences, Department of Pharmacognosy, University of Graz, Graz, Austria; Institute for Applied Botany and Pharmacognosy, University of Veterinary Medicine, Vienna, Austria; Institute of Pharmaceutical Sciences/Pharmaceutical Chemistry, University of Graz, Graz, Austria; Institute of Applied Synthetic Chemistry, TU Wien, Vienna, Austria

**Keywords:** Computational chemistry, Drug research, Natural products, Pharmacology, Phytochemistry, Synthetic chemistry

## Abstract

**Abstract:**

Inflammation is part of numerous pathological conditions, which are lacking satisfying treatment and effective concepts of prevention. A national research network project, DNTI, involving scientists from six Austrian universities as well as several external partners aimed to identify and characterize natural products capable of combating inflammatory processes specifically in the cardiovascular system. The combined use of computational techniques with traditional knowledge, high-tech chemical analysis and synthesis, and a broad range of in vitro, cell-based, and in vivo pharmacological models led to the identification of a series of promising anti-inflammatory drug lead candidates. Mechanistic studies contributed to a better understanding of their mechanism of action and delivered new knowledge on the molecular level of inflammatory processes. Herein, the used approaches and selected highlights of the results of this interdisciplinary project are presented.

**Graphical abstract:**

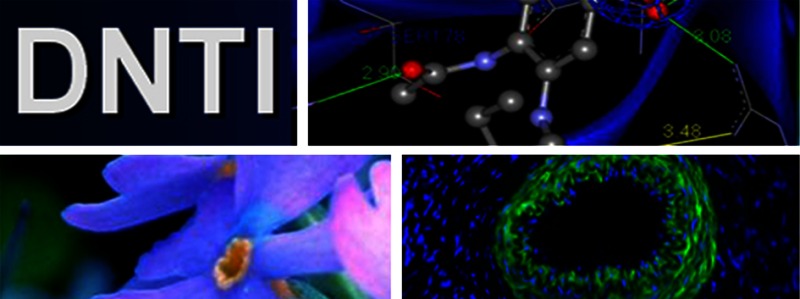

## Introduction

Although major advances have been made in understanding the cellular and molecular bases of inflammatory processes, only a limited number of optimally effective anti-inflammatory drugs are available on the market. Inflammation is involved in numerous pathological conditions, including arthritis, atherosclerosis, the metabolic syndrome, sepsis, allergies, auto-immune diseases, and cancer [1]. For most of these diseases, neither satisfying treatments nor effective concepts of prevention are available. Moreover, chronic vascular inflammatory diseases are still the main cause of death in the world and according to the World Health Organization (WHO), their incidence is expected to continue rising in the next years [2].

Inflammation is the body’s response to tissue injury, either physical (mechanical or by irradiation), by infectious agents, or by malignant or pathologically altered cells. It is associated with local cytokine production, activation of vascular cells and adhesion and transmigration of leucocytes into the site of injury. Many of the inflammatory processes elicited within vascular and immune cells are regulated by the transcription factor nuclear factor kappa B (NF-κB) [3, 4]. Major target genes of NF-κB include adhesion molecules, cytokines, growth factors, and enzymes, such as cyclooxygenase-2 (COX-2). Most prominent upstream inducers are pro-inflammatory cytokines, tumor necrosis factor-α (TNF-α), interleukin-1 (IL-1), and bacterial lipopolysaccharides (LPS). Since it is clear that NF-κB is pivotal for inducing an inflammatory response, counteracting pathways are of high relevance [4]. Important repressors of NF-κB activation are nuclear receptors, among those the glucocorticoid receptor (GR) [5], the peroxisome proliferator-activated receptors (PPARs) [6–8], the liver X receptor (LXR) [9, 10] and the NR4A family of nuclear orphan receptors [11]. PPARs exert their anti-inflammatory activities mainly by repressing the transactivation of other transcription factors, such as NF-κB or activator protein 1 (AP1), both involved e.g. in the induction of cell adhesion molecules, such as intercellular adhesion molecule-1 (ICAM-1) and vascular cell adhesion molecule-1 (VCAM-1) [12–14].

Natural products (NPs) have always been an important source of new drug leads [15, 16]. For decades, drug discovery was successfully fueled from natural sources. The first blockbuster was developed towards the end of the nineteenth century starting from salicylic acid, a compound from the willow tree, successfully marketed after acetylation as Aspirin^®^ [17]. Another commercially highly successful group of drugs, the statins, originating from *Aspergillus terreus* (lovastatin), marks the outgoing twentieth century [18]. Analysis of drugs approved by the US FDA between 1981 and 2010 showed that 50 % of all new chemical entities (NCEs) and 64 % of all small-molecule NCEs were NPs, NP-derived compounds, or compounds inspired by NPs [19]. Moreover, even though many pharmaceutical companies have discontinued their programs of drug discovery from natural sources and have focused mainly on high-throughput screening of predominantly combinatorial synthetic compound libraries throughout the last decades, in the year 2010 half of the 20 small-molecules launched as NCEs on the market were NPs or NP-derived compounds [19]. This underlines that even today, in the post genomic era, plants, fungi, marine organisms, and microorganisms are still important and effective sources for the development of new drugs or drug leads [15].

Therefore, the overall aim of the DNTI project was to identify and characterize (chemically and pharmacologically) NPs capable of combating inflammatory processes, specifically in the context of cardiovascular diseases.

## Scientific approach of the DNTI project

The DNTI project comprised a unique combination of strategies including the following aspects:

1. The focus on NPs representing a structural diversity, which is significantly higher than that of synthetic compound libraries [20].
2. The application of in silico tools like pharmacophore-based virtual screening of NP databases or parallel activity profiling to identify promising compounds for pharmacological evaluation, and to provide suggestions for their pharmacological mechanisms of action.
3. The exploitation of traditional knowledge about plants to select interesting candidates for phytochemical and subsequent pharmacological investigation.
4. The usage of plant metabolomics and the correlation with activity data in order to identify compounds most relevant for activity.

Objectives and goals of the project comprised:

1. Target selection with emphasis on pharmacological relevance and suitability for reliable pharmacophore modeling (structural information on the protein or respective ligands),
2. Compilation of information for the selection of promising natural material based on ethnopharmacological knowledge and based on virtually predicted structures of secondary metabolites from plants, fungi, microbes, etc., botanical and genetic authentication of the starting material to ensure reproducibility of the results by identifying confusions, admixtures, contaminants or (endo)symbionts,
3. Identification of bioactive NPs using either a computational or a bioassay-guided isolation approach for a target-oriented or effect-driven discovery process,
4. Pharmacological profiling and mechanistic studies on the molecular level of selected bioactive constituents by multidisciplinary research activities (in vitro, in vivo, in silico assessment),
5. Analytical studies, quality assessment of natural material, and biotechnological support, providing a highly standardized supply of natural material as sources for new lead compounds,
6. Biometric evaluation of candidates via metabolomics, and
7. Generation of semisynthetic derivatives of natural hit compounds for assessing possible improvements and synthetic up-scaling of leads.

**Fig. 1:**
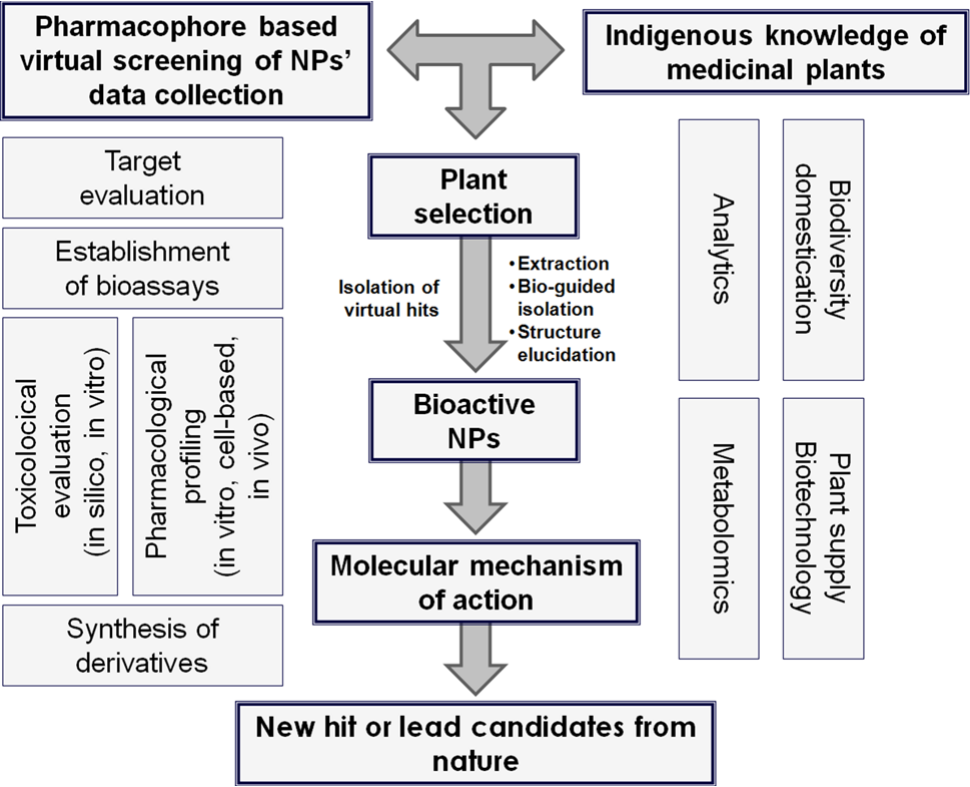
Workflow of the DNTI project

The workflow of the project is outlined in Fig. 1.

The project was structured into two periods of 3 years each. The first period (2008–2011) was devoted mainly to screening and the identification of novel NPs with pronounced anti-inflammatory effects. Therefore, at the beginning of the project, the consortium focused largely on target selection, the compilation of information for the selection of promising natural material, and the identification of bioactive NPs using either the computational or the bioassay-guided approach.

The second period (2011–2014) focused more on detailed pharmacological and toxicological profiling and in-depth characterization of interesting NPs, including the determination of the mechanisms of action of those compounds that showed promising in vitro and in vivo activity. In addition to pharmacological profiling and mechanistic studies on the molecular level of selected bioactive constituents, the second period also covered analytical studies, quality assessment of natural materials, organic synthesis and biometric identification of candidates using metabolomics. Most promising leads and their isolated or semisynthetic derivatives were thoroughly investigated in terms of their anti-inflammatory and in vitro toxicological profile.

The comprehensive and multi-facetted research approach required an interdisciplinary consortium of scientists with complementary expertise in the fields of computational, analytical, NP, and synthetic chemistry, in vitro pharmacology, cellular signaling, in vivo pharmacology, biotechnology, molecular botany, and plant conservation as well as cultivation. Thus, the project involved eight groups from six different Austrian universities in Innsbruck, Vienna, and Graz and several external partners. The highly interdisciplinary team avoided duplication of efforts and capitalized on the complementary scientific expertise of the partners, their strong background and previous contributions in the field. Ideas, reagents, new technologies, and personnel were transferred among the participating laboratories and ultimately allowed for a comprehensive analysis of the studied scientific topic.

## Organization of the DNTI project

Based on the key expertise of the different partners and due to management reasons and organizational matters, the DNTI project was organized in nine project parts (PPs), i.e., PP1–PP9 (Table 1).

**Table 1:** Overview of the project parts (PPs)

PP number	PP title	PP leader
PP1	Coordination	H. Stuppner
PP2	Identification of bioactive natural products by virtual screening	T. Langer G. Wolber D. Schuster
PP3	From in silico plant selection to bioactive natural products	H. Stuppner
PP4	From cell-based assays to molecular mechanisms	V. Dirsch
PP5	From ethnomedicine to bioactive natural products via bioassay-guided isolation	R. Bauer
PP6	Reemerging medicinal plants: bioactivity and biotechnology	B. Kopp
PP7	Medicinal plants: molecular characterization and domestication	C. Franz
PP8	In vitro and in vivo models of inflammation	B. Binder^†^ V. Bochkov
PP9	Lead-modification of medicinal plant constituents and synthetic lead up-scaling	M. Mihovilovic

^†^B. Binder—deceased

The major aim of PP1 (“Coordination”) was to ensure the success of the overall network project by a smooth, transparent, and effective management. Tasks were to set up the collaboration facilities, to drive the overall research direction of the project, and to coordinate the individual PPs.

PP2 (“Identification of bioactive NPs by virtual screening”) was devoted to the generation, application, and refinement of extended computer-assisted methods, such as pharmacophore models, for efficiently identifying NPs with potential anti-inflammatory effects and to predict bioactivities of natural compounds by virtual parallel screening. Within PP2, a pharmacophore model collection for a series of targets from the arachidonic acid cascade (e.g., cytosolic phospholipase A_2_ (cPLA_2_), microsomal prostaglandin E_2_ synthase-1 (mPGES-1), soluble epoxide hydrolase (sEH), and COX), the NF-κB pathway [e.g., inhibitor of nuclear factor kappa-B kinase-β (IKK-β)], and the nuclear receptor family (e.g., PPARα, PPARγ, and PPARβ/δ, retinoid X receptor (RXR), farnesoid X receptor (FXR), and LXR) was compiled. The models were experimentally validated and applied to virtually screen NP databases in order to predict promising NPs for isolation and pharmacological investigation. Parallel pharmacophore-based profiling of selected NPs suggested anti-inflammatory mechanisms of action for these compounds. Furthermore, bioactive NPs were docked into the respective ligand binding sites to rationalize compound binding and structure-activity relationships. Additional virtual screening methods (shape-based search, 2D similarity search) were applied as complementary approaches to identify active NPs and suggest possible targets including antitargets that could be responsible for adverse effects. PP2 played a crucial role especially in the first steps of this research project, since it produced initial data for the rational selection of NPs to be isolated and biologically tested. In addition, in silico predictions performed within PP2 guided rationalized synthesis of NP derivatives.

PP3 (“From in silico plant selection to bioactive NPs”) aimed to identify anti-inflammatory compounds from natural sources by implementation of virtually predicted ligand-target interactions obtained within PP2 in the pharmacognostic workflow. PP3 provided 3D multiconformational molecule databases containing structures of NPs for the virtual screening process. Hit candidates with predicted interactions with the binding sites of selected targets were evaluated and ranked according to chemical stability, non-toxicity, drug likeness, accessibility, and others. Selected hit candidates were isolated from appropriate natural sources and forwarded to PP4, PP5, and PP8 for assessment of the predicted pharmacological effects. Moreover, hints from traditional medicine were used as a rationale for the selection of natural material worth to be phytochemically investigated using bioassay-guided isolation strategies. Furthermore, proton nuclear magnetic resonance (^1^H-NMR) spectroscopy based metabolomics was applied (a) to identify bioactive constituents of multi-component mixtures providing a rationale for their traditional use, (b) to give insight into the molecular mechanism of action, and overall (c) to discover novel natural lead structures.

The main tasks of PP4 (“From cell-based assays to molecular mechanisms”) were (a) to provide pharmacological assays for the bioassay-guided fractionation in order to identify anti-inflammatory NPs and (b) the in-depth pharmacological characterization of hit compounds obtained either from the virtual or the bioassay-guided approach in appropriate cellular systems. Therefore, within PP4, target-oriented (e.g., for activation of PPARα, PPARβ/δ, PPARγ, LXRα, LXRβ, RXRα, and for inhibition of NF-κB and IKK-β) and functional cellular models (e.g., quantification of adhesion molecule expression in endothelial cells, glucose uptake and lipid accumulation by adipocytes, vascular smooth muscle cell (VSMC) proliferation and migration, nitric oxide (NO) release from endothelial cells) were established and provided to pharmacologically evaluate plant extracts and fractions, pure isolated compounds or synthetic derivatives of NPs as well as virtually predicted modulators, derived from the ethnopharmacological approach (PP3, PP5, PP6), the computational approach (PP2, PP3), and total synthesis (PP9). Furthermore, in-depth pharmacological characterization of compounds that have shown in vitro and in vivo activity was carried out in order to determine their mechanism of action.

Within PP5 (“From ethnomedicine to bioactive NPs via bioassay-guided isolation”), plant material especially from traditional Chinese medicine (TCM), which has been traditionally used for anti-inflammatory purposes, was selected and provided. Phytochemical characterization (metabolic profiling), bioassay-guided isolation (in close collaboration with PP4 and PP8), and structural elucidation of active constituents were the major tasks. For additional pharmacological profiling, both cellular (inhibition of leukotriene biosynthesis in human granulocytes and human platelets, inhibition of inducible nitric oxide synthase (iNOS) in macrophages) and enzymatic (inhibition of prostaglandin biosynthesis by COX-1 and COX-2) in vitro assays were performed. Moreover, metabolomics data of selected extracts obtained by liquid chromatography coupled with high-resolution-mass spectrometry (LC-HRMS) and ^1^H-NMR spectroscopy were correlated with anti-inflammatory activity by means of multivariate data analysis to identify active principles.

During the course of PP6 (“Reemerging medicinal plants: bioactivity and biotechnology”), an extensive literature survey for anti-inflammatory plant species without known mode of action was conducted at the beginning of the project. A large number of plant samples were collected in the field and botanically identified. In close cooperation with PP4 and PP8, a screening of crude extracts was performed using cell-based or enzymatic assays. Promising extracts were chosen for characterization and bioassay-guided fractionation, leading to the isolation of active compounds. In addition, data of known NPs, e.g. from the VOLKSMED database [21], were made available for in silico screening. Moreover, the production of plants, especially endangered species and species that are difficult to propagate, was accomplished through biotechnology, including establishment of gene banks, genotype selection, micropropagation of elite plants, and organ cultures.

The most important input of PP7 (“Medicinal plants: molecular characterization and domestication”) was in the fields of plant biodiversity and biosynthesis of NPs. PP7 provided molecular support for the identification and genetic improvement of plants containing novel NPs. Major tasks were authentication of plant material, marker assisted (chemotype) selection of promising plants containing new NPs, and development of conservation strategies for endangered species. Moreover, defined plant material was produced by controlled cultivation and provided for further investigations.

Within PP8 (“In vitro and in vivo models of inflammation”), cellular assays that are complimentary to those provided within PP4 and PP5 were established. To evaluate anti-inflammatory effects of plant extracts and plant-derived compounds, in vitro models to determine the induction of inflammatory cytokines such as interleukin-8 (IL-8) and adhesion molecules such as E-selectin in endothelial cells treated with the inflammatory cytokine TNF-α or the bacterial product LPS were used. Within this PP, the activation of nuclear receptors such as LXR and FXR was also addressed. In addition, PP8 was devoted to mechanistic studies as well as the investigation of the effectiveness of selected pure NPs in models of acute and chronic diseases, including chronic cardiovascular inflammation in mice. Therefore, in vivo models such as the murine femoral artery cuff model and the murine thioglycollate induced peritonitis model were used.

Starting from interesting hit candidates, the key tasks of PP9 (“Lead-modification of medicinal plant constituents and synthetic lead up-scaling”) were (a) to develop synthetic routes to some target structures in order to provide sufficient quantities for detailed pharmacological characterization and (b) based on the development of modular synthetic routes, to enable the design of focussed compound libraries in order to assess possible improvements of the initially discovered biological activities. Therefore, within PP9, structural analogs of plant constituents with recognized biological activity were synthesized and up-scaling of the synthetic processes of promising bioactive compounds was established.

During the course of DNTI, several external partners from various countries were also involved in the project. They had different tasks and focuses and supported the network among others by (a) providing purified individual compounds identified as potential hits by the in silico approach, (b) contributing to the pharmacological and toxicological evaluation of selected NPs with biological assays which were not established within the consortium, and (c) supplying plant material for further investigations.

**Fig. 2:**
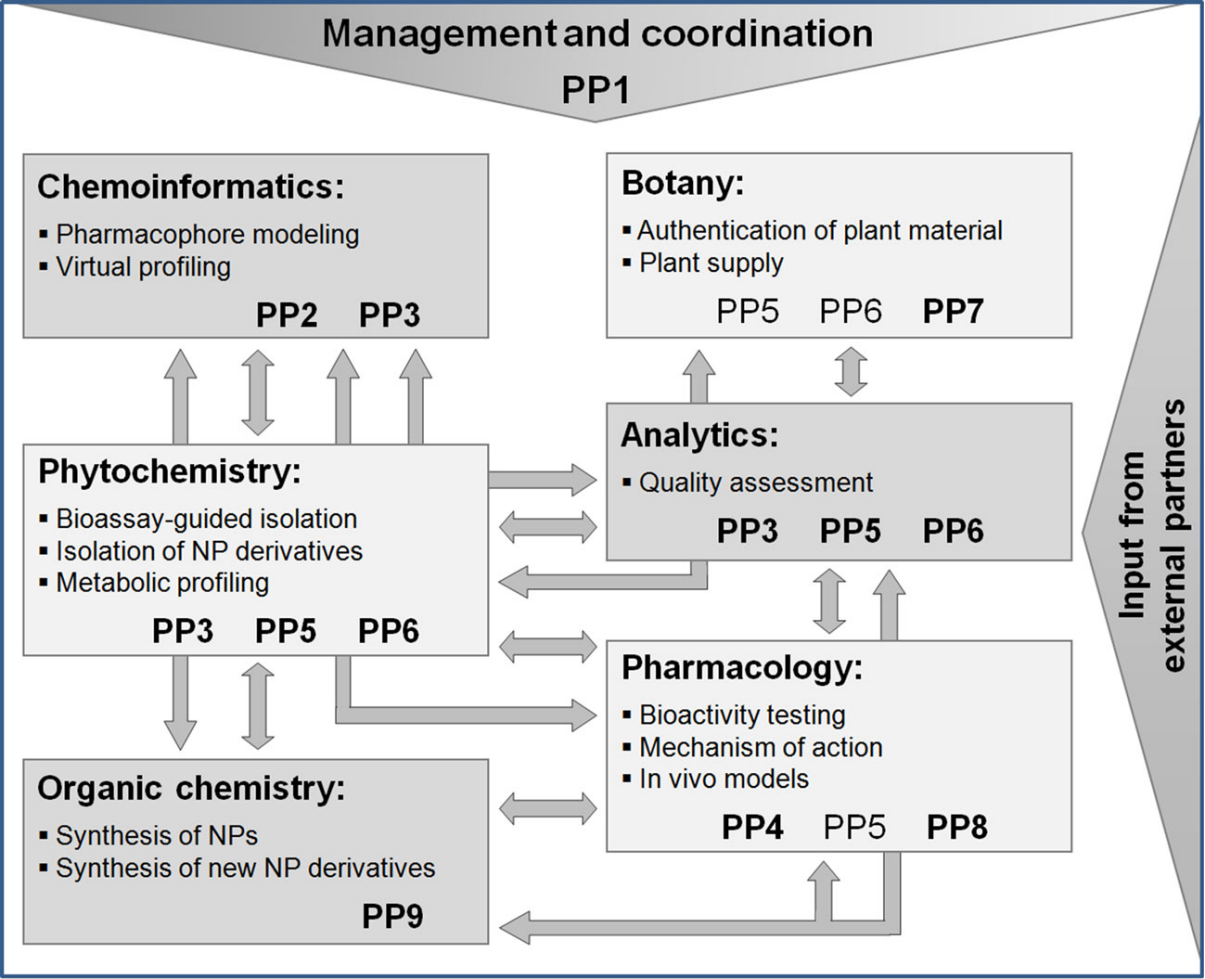
Overview of research areas including interfaces and involved PPs. PPs written in *bold font* had a major impact, *not bold* ones had a minor impact in the respective area

The manifold interfaces between the research areas and the involved PPs contributed in a synergistic way to the different aspects of the respective fields and are shown in Fig. 2. Detailed information on the network project is provided on the homepage http://www.uibk.ac.at/pharmazie/pharmakognosie/dnti/.

## Selected findings of the DNTI project

The overall aim of the DNTI project to identify and characterize anti-inflammatory NPs was addressed by a unique combination of strategies. By utilizing computational and ethnopharmacological approaches or a combination thereof and by the collaboration of experts from different research fields, NPs with highly promising in vivo and/or in vitro anti-inflammatory activity were identified, which have the potential to serve as novel lead compounds for drug development. Some of the major findings are:

### Plumericin from *Himatanthus sucuuba*

**Fig. 3:**
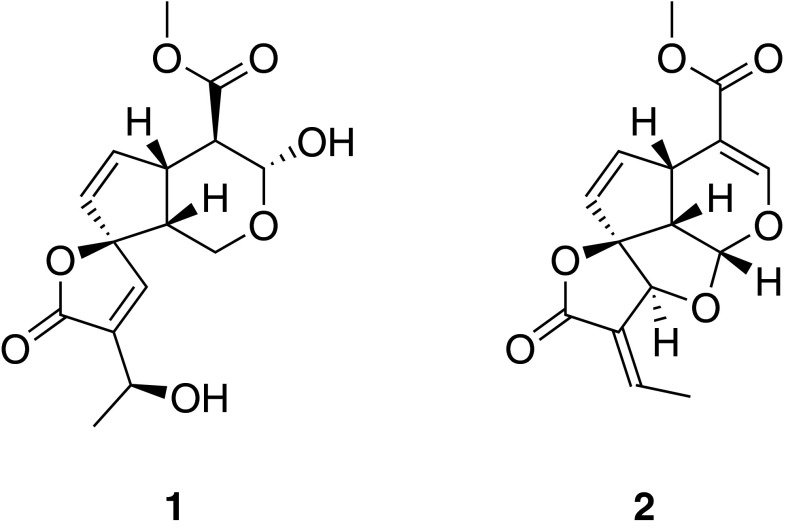
Chemical structures of plumeridoid C (**1**) and plumericin (**2**)

*Himatanthus sucuuba* (Spruce) Woodson is used in South American folk medicine to treat inflammatory diseases [22]. Although fractions have been shown to possess anti-inflammatory activity in vivo [23], the molecular mechanism of action and the bioactive principles were unknown. Bioassay-guided fractionation of *H. sucuuba* bark material led to the isolation of iridoids, flavonoids, and the lignan pinoresinol. The absolute structure of plumeridoid C (**1**) was resolved by X-ray single crystal structure analysis [24]. Plumericin (**2**) was identified as an active principle of *H. sucuuba* with potent anti-inflammatory activity. It inhibited NF-κB-mediated transactivation of a luciferase reporter gene with an *IC*_50_ of 1.1 µM and blocked TNF-α-induced expression of the adhesion molecules ICAM-1, VCAM-1, and E-selectin in endothelial cells. Further mechanistic studies were performed on **2**, demonstrating that this new scaffold NF-κB inhibitor exerts its NF-κB-inhibitory effect mainly by suppression of inhibitor of kappa B (IκB) phosphorylation and degradation and also by directly targeting IKK-β. Moreover, **2** suppressed thioglycollate-induced peritonitis in mice. Due to its exceptional potency and in vivo efficacy **2** may be ranked among the most interesting NPs described to date as NF-κB pathway blockers [25] (Fig. 3).

### Neolignans from* Magnolia officinalis*

A pharmacophore-based virtual screening of 3D NP databases resulted in the identification of neolignans as putative PPARγ agonists. This was verified by testing the neolignan magnolol (**3**), isolated from the TCM plant *Magnolia officinalis* Rehd. et Wils., and its analogues dieugenol (**4**) and tetrahydrodieugenol (**5**) for effectiveness in an in vitro PPARγ receptor binding assay. The neolignans bound to the PPARγ ligand binding domain with EC_50_ values in the nanomolar range, showing binding patterns highly similar to the clinically used PPARγ agonist pioglitazone. Compounds **4** and **5** selectively activated human PPARγ-, but not human PPARα- or PPARβ/δ-mediated luciferase reporter expression in intact cells. The observed pattern suggested partial PPARγ agonism, which is highly favourable [26].

The structurally closely related neolignan honokiol (**6**) from *M. officinalis* was virtually predicted to bind to the PPARγ ligand binding pocket as a dimer. Pharmacological investigation in a luciferase reporter assay and with purified PPARγ revealed that **6** acts as partial PPARγ agonist. Further investigations, i.e., stimulation of glucose uptake in adipocytes and adipogenic differentiation in 3T3-L1 pre-adipocytes and mouse embryonic fibroblasts, revealed that **6** stimulates basal glucose uptake similar to pioglitazone, but without inducing adipogenesis, an effect observed with pioglitazone. Furthermore, in vivo studies using diabetic KKAy mice demonstrated that oral application of **6** prevents hyperglycemia and suppresses weight gain. This outstanding non-adipogenic profile suggests **6** as promising novel pharmaceutical lead to counteract metabolic disease, and provides a molecular explanation for the usage of *M. officinalis* in traditional medicine [27].

**Fig. 4:**
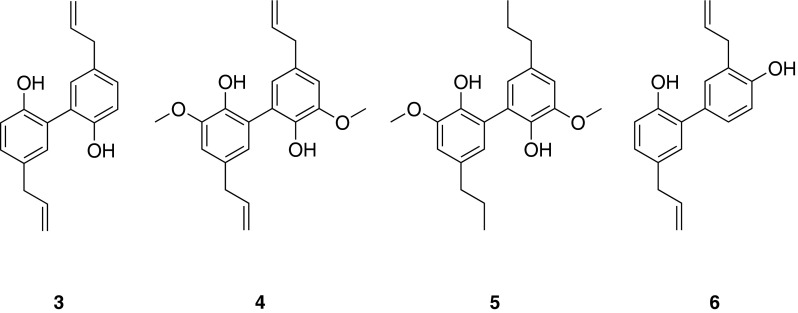
Chemical structures of magnolol (**3**), dieugenol (**4**), tetrahydrodieugenol (**5**), and honokiol (**6**)

For subsequent biological studies, a modular synthetic strategy was developed towards structural analogs of **3** and **6** based on cross coupling chemistry [28] (Fig. 4).

### Leoligin from Edelweiss (*Leontopodium nivale* ssp. *alpinum*)

The lignan leoligin (**7**), isolated from the subaerial plant parts of Edelweiss [*Leontopodium nivale* ssp. *alpinum* (Cass.) Greuter], was identified as an inhibitor of the proliferation of human umbilical vein endothelial cells (HUVECs) as well as human VSMCs. Moreover, it was shown to potently inhibit neo-intima formation in an in vivo bypass model [29]. Compound **7** was also virtually profiled and subsequently experimentally assessed for activation of the cholesteryl ester transfer protein (CETP) [30]. Additionally, potential drug–drug interactions via the cytochrome P450 enzyme system were predicted. Compound **7** was experimentally shown to be a potent CYP3A4 inhibitor, while CYP1A2 and CYP2C9 were only weakly inhibited [31].

Moreover, a screening for compounds able to stimulate angiogenesis led to 5-methoxyleoligin (**8**), a natural derivative of **7**. Pharmacological analyses revealed that in contrast to **7**, compound **8** stimulated endothelial tube formation, angiogenic sprouting, and angiogenesis in a chicken chorioallantoic membrane assay. Furthermore, in an in vivo rat myocardial infarct model, **8** increased cardiac performance, decreased apoptosis of cardiomyocytes, induced arteriogenesis but not angiogenesis in the infarction area, and diminished the loss of myocardial muscle. This outstanding pharmacological activity renders **8** one of if not the first low molecular weight compound that may lead to an improvement of myocardial function after myocardial infarction [32].

**Fig. 5:**
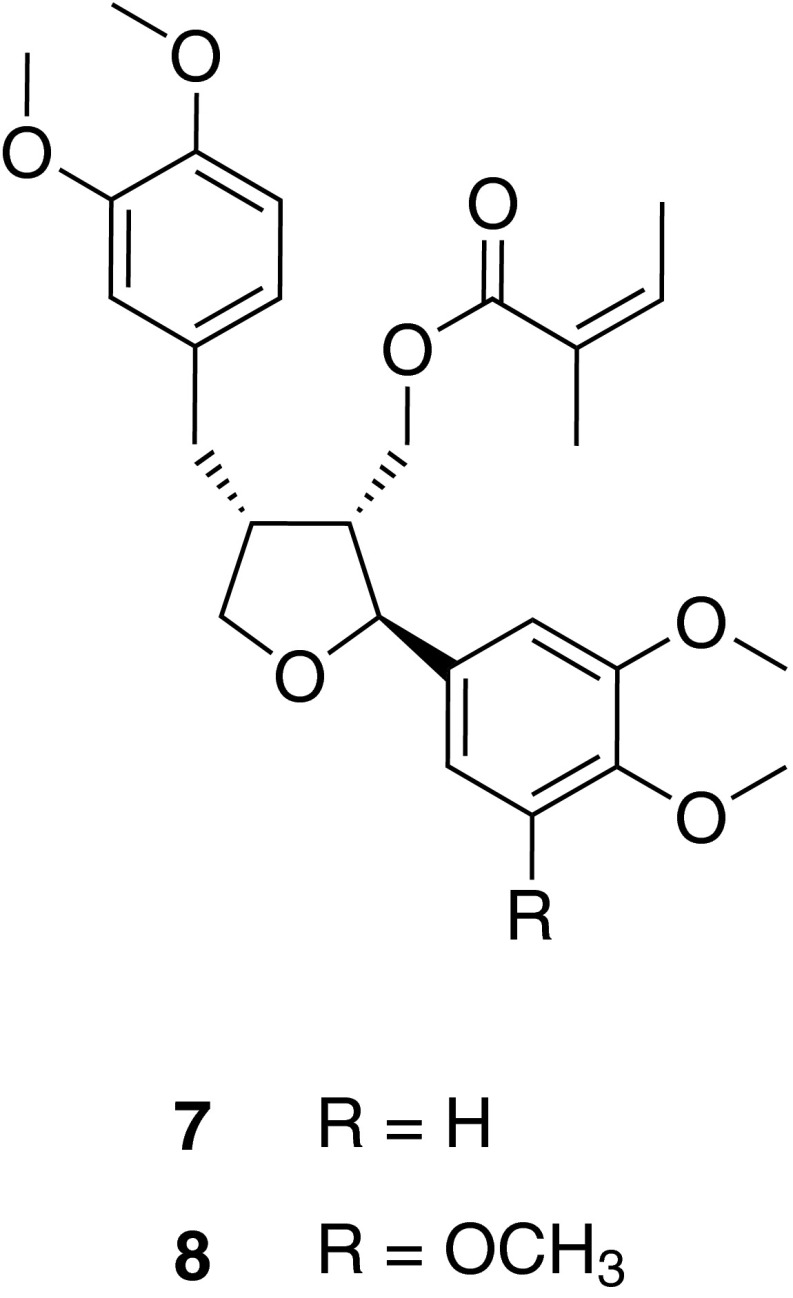
Chemical structures of leoligin (**7**) and 5-methoxyleoligin (**8**)

In order to provide the natural lignan derivatives in sufficient amounts, the production of lignans in hairy root cultures of *L. nivale* ssp. *alpinum* was investigated under different cultivation conditions (elicitation with silver nitrate, sucrose, and others) leading to contents of the pharmacologically active lignans **7** and **8** comparable to those found in Edelweiss from field cultivation [33]. Alternatively, total synthesis of **7** was achieved in stereoselective fashion [34]. Key reaction steps of the synthetic route involved lipase mediated kinetic resolution to establish chirality, radical cyclization towards the tetrahydrofuran core ring, as well as a hydroboration-coupling strategy in order to enable diversity oriented design of a compound library of derivatives of **7** (and **8**) (Fig. 5).

### Depsides and depsidones from lichen species

**Fig. 6:**
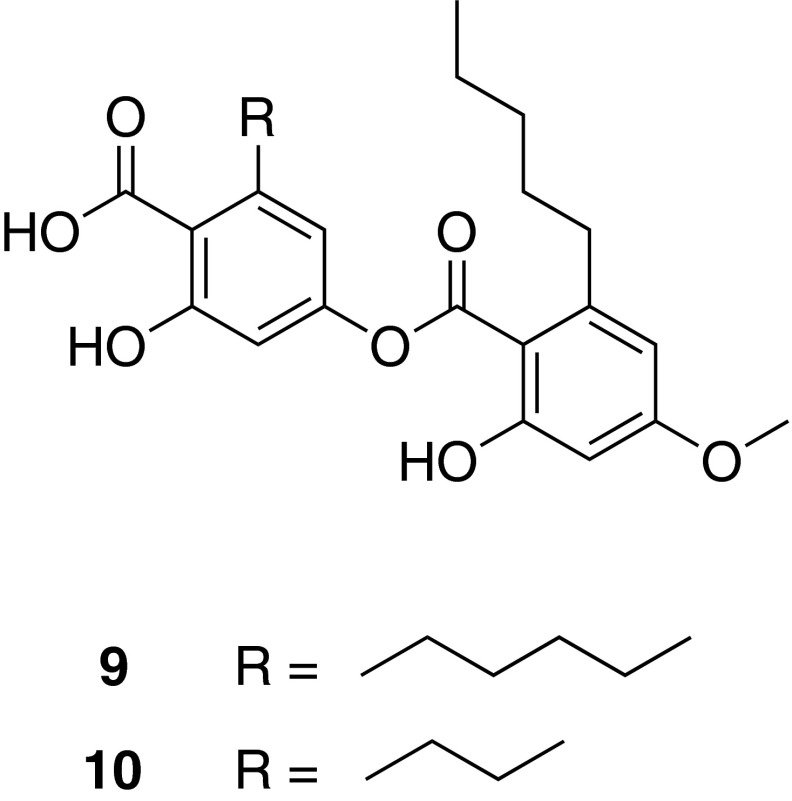
Chemical structures of perlatolic acid (**9**) and imbricaric acid (**10**)

Specific inhibition of mPGES-1, an inflammation induced enzyme, is assumed not to affect the biosynthesis of physiologic prostaglandin (PG)E_2_ and other COX-derived prostanoids. Therefore, inhibitors of mPGES-1 are expected to possess anti-inflammatory activity without leading to unwanted side effects typically associated with COX inhibitors. Aiming to identify novel natural inhibitors of this innovative target, pharmacophore models were developed and experimentally validated [35]. Virtual screening of NP databases with these pharmacophore models led to the identification of depsides and depsidones from lichen species as potent mPGES-1 inhibitors [36]. Among them, perlatolic acid (**9**) and imbricaric acid (**10**) from *Cetrelia monachorum* (Zahlbr.) Culb. et Culb. showed strong inhibition of mPGES-1 (*IC*_50_ = 0.4 and 1.9 µM, respectively, on the purified enzyme), 5-lipoxygenase (5-LO) (*IC*_50_ = 1.8 and 5.3 µM, respectively, in a cell-based assay and *IC*_50_ = 0.4 and 3.5 µM, respectively, on the purified enzyme), as well as of TNF-α-induced NF-κB activation (*IC*_50_ = 7.0 and 2.0 µM, respectively, in luciferase reporter cells). The anti-inflammatory effect of **9** was further confirmed in a murine in vivo model of inflammation, in which a significant reduction of thioglycollate-induced recruitment of leukocytes to the peritoneum was demonstrated [37] (Fig. 6).

### Benzofurans from *Krameria lappacea*

*Krameria lappacea* (Dombey) Burdet et Simpson roots are used in traditional medicine for the treatment of oropharyngeal inflammation. The dichloromethane extract of the root material as well as eleven isolated lignan derivatives exhibited a prominent anti-edema effect in a croton-oil induced mouse ear edema model, comparable to the effect of the positive control indomethacin. Pharmacological investigations of the isolated NPs in different in vitro models revealed the inhibition of several molecular targets by the isolated compounds, i.e., NF-κB, COX-1 and -2, 5-LO, and mPGES-1, as well as antioxidant effects [38]. Interestingly, among the eleven isolated compounds, only 2-(2,4-dihydroxyphenyl)-5-(*E*)-propenylbenzofuran (**11**) enhanced endothelial nitric oxide synthase (eNOS) activity and NO release in endothelial cells [39]. To quantify the active lignan derivatives in the roots as well as in Tinctura Ratanhiae, a HPLC method was developed and validated. Analyses of several root and tincture samples showed that (+)-conocarpan (**12**) and ratanhiaphenol II (**13**) are the major compounds, respectively [40].

**Fig. 7:**
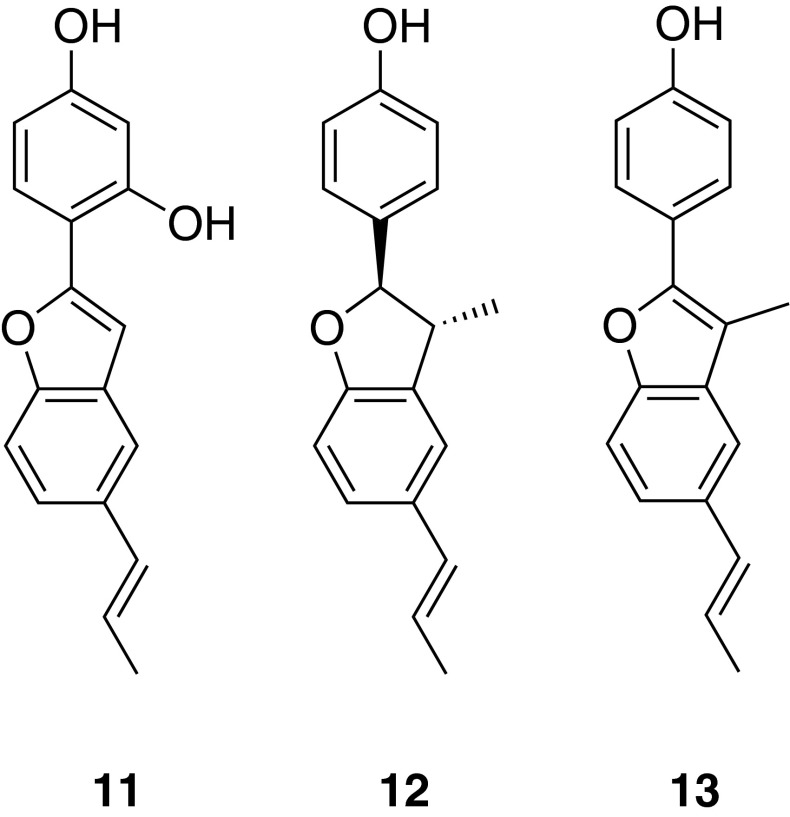
Chemical structures of 2-(2,4-dihydroxyphenyl)-5-(*E*)-propenylbenzofuran (**11**), (+)-conocarpan (**12**), and ratanhiaphenol II (**13**)

Synthetic access to benzofurane-type analogs of the natural compound isolates was achieved by CH-activation [41]. This is a particularly valuable chemical transformation, as it does not require pre-activation of particular reaction centers but rather exploits intrinsic reactivity differences of C–H bonds in organic structures [42] (Fig. 7).

### Isosilybin A from *Silybum marianum*

**Fig. 8:**
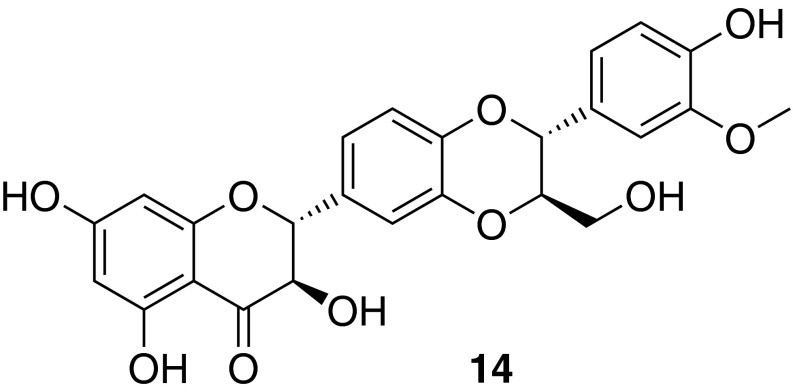
Chemical structure of isosilybin A (**14**)

Silymarin represents a concentrated phenolic mixture from milk thistle [*Silybum marianum* (L.) Gaertn.] fruits, and is traditionally used in the treatment of a variety of liver diseases [43]. There is a growing body of evidence supporting a role for PPARs in the pathogenesis of nonalcoholic fatty liver disease [44]. Therefore, considering the relevant traditional use of silymarin, as well as the existing potential to modulate liver disease by PPAR modulation, it was studied whether silymarin and its purified flavonolignan and flavonoid constituents were able to activate PPARγ. Phytochemical analysis revealed as main silymarin constituents silybin A, silybin B, isosilybin A (**14**), isosilybin B, silychristin, silydianin, and taxifolin. These compounds were evaluated for their ability to cause transactivation of a PPARγ-dependent luciferase reporter gene. As a result of this study, **14** was identified as the first PPARγ-agonist possessing a flavonolignan-type scaffold [45] (Fig. 8).

### Polyacetylenes from *Notopterygium incisum*

**Fig. 9:**
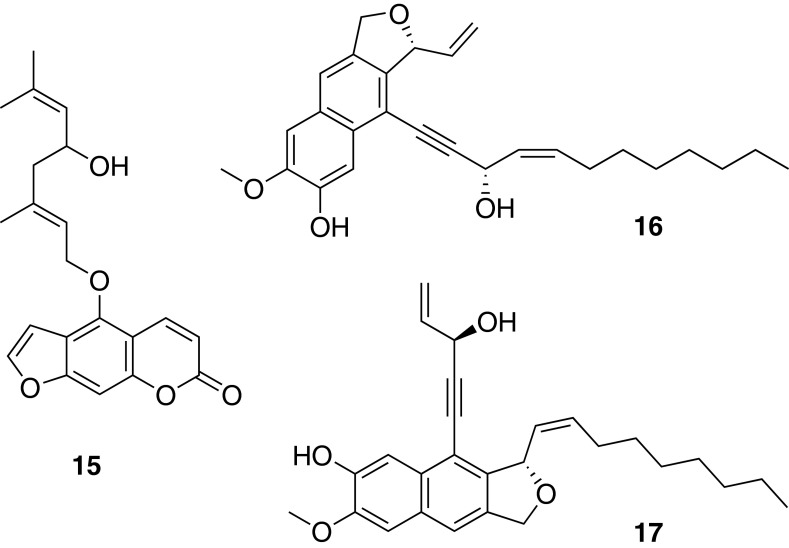
Chemical structures of E-notopterol (**15**), notoincisol B (**16**), and notoincisol C (**17**)

The dichloromethane extract of the roots of *Notopterygium incisum* Ting ex Chang activated PPARγ and showed strong anti-inflammatory activity in vitro. Bioassay-guided fractionation resulted in the isolation of numerous compounds from different classes (coumarins, furanocoumarins, sesquiterpenoids, ferulic acid derivatives, polyacetylenes, and polyacetylene adducts). The crystal structure of the racemic coumarin E-notopterol (**15**) was newly established [46]. While the coumarins and furanocoumarins were inactive, the polyacetylenes showed activities in the iNOS assay with *IC*_50_ values in the range of 10–30 µM [47]. Furthermore, the polyacetylenes isolated from *N. incisum* were identified as a novel class of specific partial PPARγ agonists [48]. In addition, a series of polyacetylene hybrid molecules with sesquiterpene or phenylpropane units were isolated and structurally identified by means of NMR and HRMS. Notoincisol B (**16**) and notoincisol C (**17**) represent two new skeletons. From this series of compounds, notoethers A–C, notoincisol A, as well as **16** showed PPARγ agonistic effects [49]. A specific DNA barcoding method was developed to authenticate *N. incisum* and to differentiate it from other *Notopterygium* sp. and related Apiaceae [47] (Fig. 9).

### Indirubin-3′-monoxime, a derivative of the dye indirubin

**Fig. 10:**
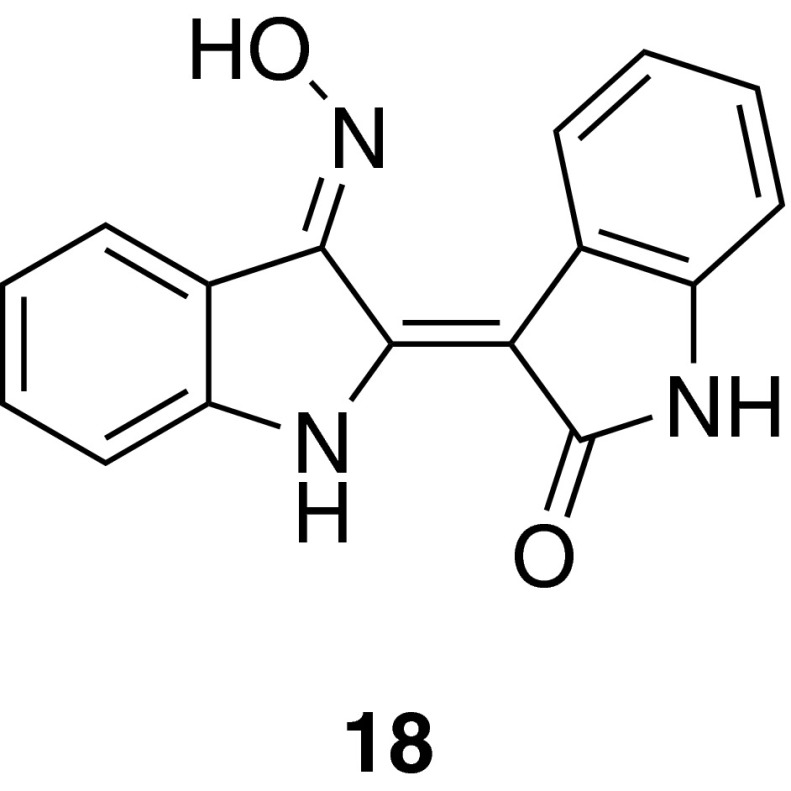
Chemical structure of indirubin-3′-monoxime (I3MO) (**18**)

The alkaloid indirubin-3′-monoxime (I3MO) (**18**) is a derivative of the red dye indirubin, which is a component of the TCM Danggui Longhui Wan, and has demonstrated encouraging clinical results in chronic myelocytic leukemia patients [50]. Compound **18** revealed that it is able to inhibit VSMC proliferation in vitro and neointima formation in vivo in a murine femoral artery cuff model of restenosis. On the cellular level, **18** specifically inhibited signal transducer and activator of transcription 3 (STAT3) phosphorylation in VSMCs, which was shown to contribute to the inhibition of cell proliferation [51]. 12/15-LO has been identified as a crucial mediator in platelet-derived growth factor (PDGF)-triggered STAT3 activation, which is targeted by **18** [52] (Fig. 10).

### Compounds from traditional Austrian medicinal plants

**Fig. 11:**
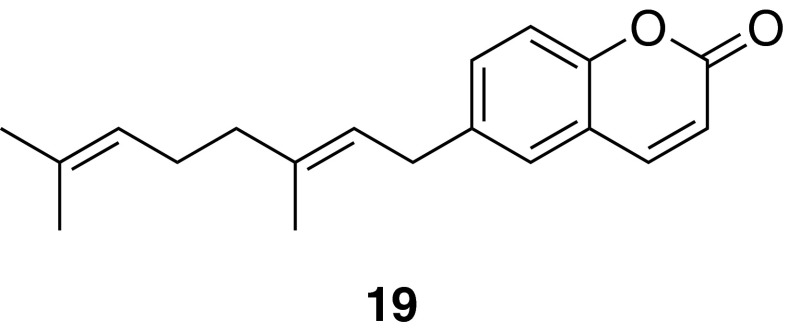
Chemical structure of ostruthin (**19**)

The VOLKSMED database comprises ethnopharmacological knowledge about plants native to Austria and its adjacent regions. Extracts of 71 herbal drugs traditionally used in Austrian folk medicine (retrieved from the VOLKSMED database) were evaluated for their potential to inhibit NF-κB, and/or activate PPARα and PPARγ. Extracts of more than 50 plants were active in at least one of the three models [53]. For further bioassay-guided fractionation, extracts were considered that had potent activity and were active in several in vitro and cellular models. An extract of *Melampyrum pratense* L. (Koch) was identified to both stimulate PPARs and inhibit the activation of NF-κB. Bioassay-guided fractionation of this plant extract identified several active flavonoids and iridoids including melampyroside and mussaenoside and the phenolic compound lunularin, which were able to suppress NF-κB and its target genes IL-8 and E-selectin to different extents [54]. Another interesting herbal drug from the VOLKSMED database that was studied is the rhizome of *Peucedanum ostruthium* (L.) Koch (masterwort), which has been traditionally used in the Alpine region as an ingredient in liqueurs and bitters. To clearly identify *P. ostruthium* and to detect common adulterants in the crude drug, a high-resolution melting curve analysis was performed [55]. A sensitive and specific HPLC–DAD–MS method was developed for the simultaneous identification and quantification of the main coumarin constituents of this herbal drug [56]. A dichloromethane extract of the rhizome of *P. ostruthium* was found to inhibit VSMC proliferation, and bioassay-guided fractionation led to the identification of ostruthin (**19**) as the active component mediating this effect [57] (Fig. 11).

### Compounds from traditional Vietnamese medicinal plants

In a pharmacological screening approach, 42 extracts of 18 plants used in traditional Vietnamese medicine for the treatment of inflammatory disease conditions were investigated on their ability to inhibit NF-κB and activate nuclear factor-erythroid 2-related factor 2 (Nrf2). Based on the screening results, *Oroxylum indicum* (L.) Kurz, *Eurycoma longifolia* Jack, and *Chromolaena odorata* (L.) King et Rob. were selected for further investigations.

Phytochemical studies on the active dichloromethane extract of the bark of *O. indicum* led to the isolation of ten flavonoids. The flavonoid aglycones oroxylin A (**20**), chrysin (**21**), hispidulin (**22**), and baicalein (**23**) inhibited NF-κB with *IC*_50_ values of 3.9, 7.2, 9.0, and 28.1 µM, while the corresponding glycosides showed no inhibitory activity at a concentration of 30 µM [58].

Bioassay-guided fractionation of the methanolic extract of *E. longifolia* roots using an NF-κB-driven luciferase reporter gene assay resulted in the identification of a novel quassinoid, eurycomalide C (**24**), and 27 known compounds including 11 quassinoids, six alkaloids, two coumarins, a squalene derivative, a triterpenoid, and six phenolic compounds. C_19_- and C_20_-type quassinoids, canthin-6-one alkaloids, and the alkaloid β-carboline (**25**) were identified as potent NF-κB inhibitors with *IC*_50_ values in the low micromolar range. The quassinoid eurycomalactone (**26**) with an *IC*_50_ of 0.5 µM was identified as the most potent NF-κB inhibitor. In the group of alkaloids, 9-hydroxycanthin-6-one (**27**) possessed the strongest activity (*IC*_50_ = 3.8 µM) [59].

**Fig. 12:**
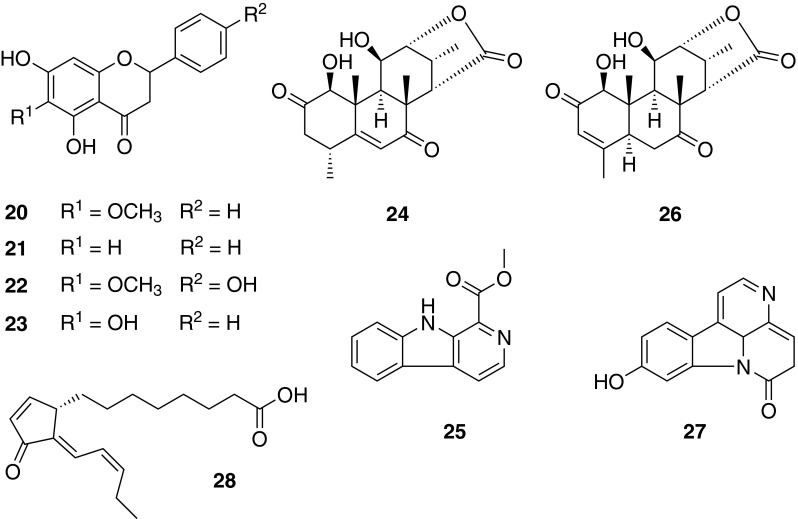
Chemical structures of oroxylin A (**20**), chrysin (**21**), hispidulin (**22**), baicalein (**23**), eurycomalide C (**24**), β-carboline (**25**), eurycomalactone (**26**), 9-hydroxycanthin-6-one (**27**), and chromomoric acid C-I (**28**)

The dichloromethane extract of *C. odorata* leaves displayed the most potent activity in the NF-κB as well as the Nrf2 assay. Bioassay-guided fractionation led to the isolation of several phytoprostanes. Among those, chromomoric acid C-I (**28**) was identified as a strong Nrf2 activator with a concentration doubling the response of vehicle-treated cells (CD) of 5.2 μM and an inhibitory effect on NF-κB (*IC*_50_ = 6.9 µM) [60] (Fig. 12).

### Compounds from dietary spices

Piperine (**29**) and [6]-shogaol (**30**) were identified as inhibitors of VSMC proliferation. They originate from black pepper (*Piper nigrum* L.) and ginger (*Zingiber officinale* Roscoe), respectively, which are important dietary spices that are also used in the traditional medicine.

The inhibitory effect of **29** was quantified by determining the metabolic activity of PDGF-activated VSMCs by the resazurin conversion method (*IC*_50_ = 21.6 µM) and by quantifying de novo DNA synthesis by BrdU incorporation. In order to identify compounds with enhanced activity and to deduce a structure activity relationship (SAR), 10 naturally occurring and 15 synthesized derivatives of **29** were additionally analyzed. As a result, several more potent congeners of **29** were identified, in particular the naturally occurring pipertipine, and the two synthetic derivatives (2*E*,4*E*)-*N*,*N*-dibutyl-5-(3,5-dimethoxyphenyl)penta-2,4-dienamide and (*E*)-*N*,*N*-dibutyl-3-(naphtho[2,3-*d*][1,3]dioxol-5-yl)acrylamide. These derivatives displayed *IC*_50_ values of 3.4, 6.0, and 7.9 µM, respectively [61].

Chemical variation of **29** focused on three regions [62]: substitution pattern at the amide function, structural rigidification of the unsaturated linker region, and modification of the terminal aryl core. Again, a flexible and modular strategy for compound assembly was employed in particular for linker and aryl derivatization based on metal-assisted C–C bond formation reactions [63].

**Fig. 13:**
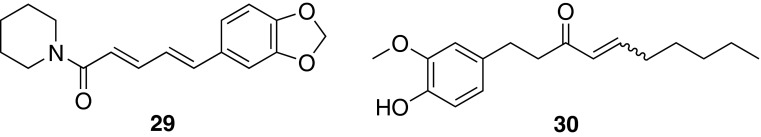
Chemical structures of piperine (**29**) and [6]-shogaol (**30**)

The inhibitory effect of **30** from *Z. officinale* was also quantified by determination of the total metabolic activity of PDGF-induced VSMCs by the resazurin conversion method (*IC*_50_ = 2.7 μM). Its activity was verified by quantification of de novo DNA synthesis by the BrdU incorporation assay (*IC*_50_ = 3.0 μM). Application of the compound also inhibited the total cellular biomass growth, quantified by crystal violet staining, with an *IC*_50_ of 1.1 μM. Four major bioactive ginger compounds, [6]-gingerol, zingerone, [6]-paradol, and rac-[6]-dihydroparadol, which were less active than **30**, were studied as well. In depth analysis of the inhibitory action of **30** uncovered accumulation of VSMCs in the G0/G1 phase of the cell cycle that was associated with the activation of the Nrf2/HO-1 pathway [64] (Fig. 13).

### Metabolic profiling of *Lonicera* and *Clematis* species

Species of the genera *Lonicera* L. and *Clematis* L. are traditionally used for the treatment of inflammation-related diseases. Based on this ethnopharmacological knowledge and the results of a pharmacological screening of a large number of extracts of plants used in TCM, anti-inflammatory species of these genera were selected for proof-of-principle studies in which profiling data obtained by LC–MS analysis and bioactivities of crude extracts were correlated to identify the active constituents [65, 66].

### Key reviews combining the multidisciplinary expertise of the DNTI participants

Making use of the gathered experience and the unique combination of multidisciplinary expertise in DNTI, two reviews were jointly developed, one focusing on NP activators of PPARγ [67] and another one reviewing established strategies for discovery and resupply of plant-derived bioactive NPs [68].

## Conclusion

Within the DNTI project, following a unique combination of strategies, a plethora of compounds from natural sources have been identified that are capable of combating inflammatory processes and may act as novel lead structures for the highly demanded anti-inflammatory therapy, especially in the field of cardiovascular diseases. This underlines that NPs represent a valuable source for lead discovery. The complexity of the project demonstrates that NPs research requires a multidisciplinary strategic approach and a complementary scientific expertise.

## References

[1] Weiss U (2008). Nature.

[2] World Health Organization (2013) A gobal brief on hypertension—Silent killer, global public health crisis. WHO reference number: WHO/DCO/WHD/2013.2 edn

[3] Verstrepen L, Beyaert R (2014). Biochem Pharmacol.

[4] Karin M, Yamamoto Y, Wang QM (2004). Nat Rev Drug Discov.

[5] Caratti G, Matthews L, Poolman T, Kershaw S, Baxter M, Ray D (2015). Clin Endocrinol (Oxf).

[6] Glass CK, Saijo K (2010). Nat Rev Immunol.

[7] Marx N, Duez H, Fruchart JC, Staels B (2004). Circ Res.

[8] Millar JS (2013). Curr Opin Lipidol.

[9] Steffensen KR, Jakobsson T, Gustafsson JA (2013). Expert Opin Ther Targets.

[10] Parikh M, Patel K, Soni S, Gandhi T (2014). J Atheroscler Thromb.

[11] McMorrow JP, Murphy EP (2011). Biochem Soc Trans.

[12] Cabrero A, Laguna JC, Vazquez M (2002). Curr Drug Targets Inflamm Allergy.

[13] Kostadinova R, Wahli W, Michalik L (2005). Curr Med Chem.

[14] Wang N, Verna L, Chen NG, Chen J, Li H, Forman BM, Stemerman MB (2002). J Biol Chem.

[15] Cragg GM, Newman DJ (2013). Biochim Biophys Acta.

[16] Harvey AL (2008). Drug Discov Today.

[17] Heinrich M, Liu H-W, Mander L (2010). 3.12—Ethnopharmacology and drug discovery. Comprehensive Natural Products II.

[18] Appendino G, Fontana G, Pollastro F, Liu H-W, Mander L (2010). 3.08—Natural products drug discovery. Comprehensive Natural Products II.

[19] Newman DJ, Cragg GM (2012). J Nat Prod.

[20] Henkel T, Brunne RM, Mueller H, Reichel F (1999). Angew Chem Int Ed.

[21] Saukel J, Kubelka W (1994). Sci Pharm.

[22] Amaral ACF, Ferreira JLP, Pinheiro MLB, Silva JRdA (2007) Phcog Rev 1:305

[23] de Miranda AL, Silva JR, Rezende CM, Neves JS, Parrini SC, Pinheiro ML, Cordeiro MC, Tamborini E, Pinto AC (2000). Planta Med.

[24] Waltenberger B, Rollinger JM, Griesser UJ, Stuppner H, Gelbrich T (2011). Acta Crystallogr C.

[25] Fakhrudin N, Waltenberger B, Cabaravdic M, Atanasov AG, Malainer C, Schachner D, Heiss EH, Liu R, Noha SM, Grzywacz AM, Mihaly-Bison J, Awad EM, Schuster D, Breuss JM, Rollinger JM, Bochkov V, Stuppner H, Dirsch VM (2014). Br J Pharmacol.

[26] Fakhrudin N, Ladurner A, Atanasov AG, Heiss EH, Baumgartner L, Markt P, Schuster D, Ellmerer EP, Wolber G, Rollinger JM, Stuppner H, Dirsch VM (2010). Mol Pharmacol.

[27] Atanasov AG, Wang JN, Gu SP, Bu J, Kramer MP, Baumgartner L, Fakhrudin N, Ladurner A, Malainer C, Vuorinen A, Noha SM, Schwaiger S, Rollinger JM, Schuster D, Stuppner H, Dirsch VM, Heiss EH (2013). Biochim Biophys Acta.

[28] Rycek L, Puthenkalam R, Schnürch M, Ernst M, Mihovilovic MD (2015). Bioorg Med Chem Lett.

[29] Reisinger U, Schwaiger S, Zeller I, Messner B, Stigler R, Wiedemann D, Mayr T, Seger C, Schachner T, Dirsch VM, Vollmar AM, Bonatti JO, Stuppner H, Laufer G, Bernhard D (2009). Cardiovasc Res.

[30] Duwensee K, Schwaiger S, Tancevski I, Eller K, van Eck M, Markt P, Linder T, Stanzl U, Ritsch A, Patsch JR, Schuster D, Stuppner H, Bernhard D, Eller P (2011). Atherosclerosis.

[31] Kaserer T, Höferl M, Müller K, Elmer S, Ganzera M, Jäger W, Schuster D (2015). Mol Inf.

[32] Messner B, Kern J, Wiedemann D, Schwaiger S, Turkcan A, Ploner C, Trockenbacher A, Aumayr K, Bonaros N, Laufer G, Stuppner H, Untergasser G, Bernhard D (2013). PLoS ONE.

[33] Wawrosch C, Schwaiger S, Stuppner H, Kopp B (2014). Fitoterapia.

[34] Mihovilovic MD, Linder T, Atanasov A, Dirsch VM, Stuppner H, Schwaiger S, Bernhard D (2015) Novel Leoligin Derivatives for Applications as Proliferation Inhibitors. Austrian Pat. Appl. AT 2015/050160

[35] Waltenberger B, Wiechmann K, Bauer J, Markt P, Noha SM, Wolber G, Rollinger JM, Werz O, Schuster D, Stuppner H (2011). J Med Chem.

[36] Bauer J, Waltenberger B, Noha SM, Schuster D, Rollinger JM, Boustie J, Chollet M, Stuppner H, Werz O (2012). Chem Med Chem.

[37] Oettl SK, Gerstmeier J, Khan SY, Wiechmann K, Bauer J, Atanasov AG, Malainer C, Awad EM, Uhrin P, Heiss EH, Waltenberger B, Remias D, Breuss JM, Boustie J, Dirsch VM, Stuppner H, Werz O, Rollinger JM (2013). PLoS On e.

[38] Baumgartner L, Sosa S, Atanasov AG, Bodensieck A, Fakhrudin N, Bauer J, Favero GD, Ponti C, Heiss EH, Schwaiger S, Ladurner A, Widowitz U, Loggia RD, Rollinger JM, Werz O, Bauer R, Dirsch VM, Tubaro A, Stuppner H (2011). J Nat Prod.

[39] Ladurner A, Atanasov AG, Heiss EH, Baumgartner L, Schwaiger S, Rollinger JM, Stuppner H, Dirsch VM (2012). Biochem Pharmacol.

[40] Baumgartner L, Schwaiger S, Stuppner H (2011). J Pharm Biomed Anal.

[41] Dao-Huy T, Haider M, Glatz F, Schnürch M, Mihovilovic MD (2014) Eur J Org Chem 2014:8119 10.1002/ejoc.201403125 PMC4502765 26213483

[42] Schnürch M (2015) Arkivoc 2015:212

[43] Polyak SJ, Ferenci P, Pawlotsky JM (2013). Hepatology.

[44] Kallwitz ER, McLachlan A, Cotler SJ (2008). World J Gastroenterol.

[45] Pferschy-Wenzig EM, Atanasov AG, Malainer C, Noha SM, Kunert O, Schuster D, Heiss EH, Oberlies NH, Wagner H, Bauer R, Dirsch VM (2014). J Nat Prod.

[46] Schinkovitz A, Belaj F, Kunert O, Bauer R (2009). Acta Crystallogr Sect E: Struct Rep Online.

[47] Blunder M, Liu X, Kunert O, Winkler NA, Schinkovitz A, Schmiderer C, Novak J, Bauer R (2014). Planta Med.

[48] Atanasov AG, Blunder M, Fakhrudin N, Liu X, Noha SM, Malainer C, Kramer MP, Cocic A, Kunert O, Schinkovitz A, Heiss EH, Schuster D, Dirsch VM, Bauer R (2013). PLoS ONE.

[49] Liu X, Kunert O, Blunder M, Fakhrudin N, Noha SM, Malainer C, Schinkovitz A, Heiss EH, Atanasov AG, Kollroser M, Schuster D, Dirsch VM, Bauer R (2014). J Nat Prod.

[50] Blazevic T, Heiss EH, Atanasov AG, Breuss JM, Dirsch VM, Uhrin P (2015) Evid Based Complement Alternat Med 2015:654098 10.1155/2015/654098 PMC4589628 26457112

[51] Schwaiberger AV, Heiss EH, Cabaravdic M, Oberan T, Zaujec J, Schachner D, Uhrin P, Atanasov AG, Breuss JM, Binder BR, Dirsch VM (2010). Arterioscler Thromb Vasc Biol.

[52] Blazevic T, Schwaiberger AV, Schreiner CE, Schachner D, Schaible AM, Grojer CS, Atanasov AG, Werz O, Dirsch VM, Heiss EH (2013). J Biol Chem.

[53] Vogl S, Picker P, Mihaly-Bison J, Fakhrudin N, Atanasov AG, Heiss EH, Wawrosch C, Reznicek G, Dirsch VM, Saukel J, Kopp B (2013). J Ethnopharmacol.

[54] Vogl S, Atanasov AG, Binder M, Bulusu M, Zehl M, Fakhrudin N, Heiss EH, Picker P, Wawrosch C, Saukel J, Reznicek G, Urban E, Bochkov V, Dirsch VM, Kopp B (2013) Evid Based Complement Alternat Med 2013:395316 10.1155/2013/395316 PMC3600302 23533479

[55] Schmiderer C, Ruzicka J, Novak J (2015). Mol Cell Probes.

[56] Vogl S, Zehl M, Picker P, Urban E, Wawrosch C, Reznicek G, Saukel J, Kopp B (2011). J Agric Food Chem.

[57] Joa H, Vogl S, Atanasov AG, Zehl M, Nakel T, Fakhrudin N, Heiss EH, Picker P, Urban E, Wawrosch C, Saukel J, Reznicek G, Kopp B, Dirsch VM (2011). J Nat Prod.

[58] Tran TV, Malainer C, Schwaiger S, Hung T, Atanasov AG, Heiss EH, Dirsch VM, Stuppner H (2015). J Ethnopharmacol.

[59] Tran TV, Malainer C, Schwaiger S, Atanasov AG, Heiss EH, Dirsch VM, Stuppner H (2014). J Nat Prod.

[60] Heiss EH, Tran TV, Zimmermann K, Schwaiger S, Vouk C, Mayerhofer B, Malainer C, Atanasov AG, Stuppner H, Dirsch VM (2014). J Nat Prod.

[61] Mair CE, Liu R, Atanasov AG, Wimmer L, Nemetz-Fiedler D, Sider N, Heiss EH, Mihovilovic MD, Dirsch VM, Rollinger JM (2015). Planta Med.

[62] Schoffmann A, Wimmer L, Goldmann D, Khom S, Hintersteiner J, Baburin I, Schwarz T, Hintersteininger M, Pakfeifer P, Oufir M, Hamburger M, Erker T, Ecker GF, Mihovilovic MD, Hering S (2014). J Med Chem.

[63] Wimmer L, Schonbauer D, Pakfeifer P, Schoffmann A, Khom S, Hering S, Mihovilovic MD (2015). Org Biomol Chem.

[64] Liu R, Heiss EH, Sider N, Schinkovitz A, Groblacher B, Guo D, Bucar F, Bauer R, Dirsch VM, Atanasov AG (2015). Mol Nutr Food Res.

[65] Ortmann S, Monschein M, Hartler J, Zhao YM, Miao JH, Thallinger GG, Bauer R (2014) Planta Med 80:P1M12

[66] Monschein M, Pferschy-Wenzig EM, Ortmann S, Huber C, Heiss E, Malainer C, Georgiev Atanasov A, Dirsch V, Hartler J, Thallinger G, Miao JH, Bauer R (2014) Planta Med 80:P1M8

[67] Wang L, Waltenberger B, Pferschy-Wenzig EM, Blunder M, Liu X, Malainer C, Blazevic T, Schwaiger S, Rollinger JM, Heiss EH, Schuster D, Kopp B, Bauer R, Stuppner H, Dirsch VM, Atanasov AG (2014). Biochem Pharmacol.

[68] Atanasov AG, Waltenberger B, Pferschy-Wenzig EM, Linder T, Wawrosch C, Uhrin P, Temml V, Wang L, Schwaiger S, Heiss EH, Rollinger JM, Schuster D, Breuss JM, Bochkov V, Mihovilovic MD, Kopp B, Bauer R, Dirsch VM, Stuppner H (2015). Biotechnol Adv.

